# Relationship between clinical and cytokines profile of brain-death donors

**DOI:** 10.1186/cc12620

**Published:** 2013-06-19

**Authors:** SL Mello, M da Consolação Vieira Moreira, RMFL Silva

**Affiliations:** 1 Faculdade de Medicina da Universidade Federa de Minas Gerais, Pampulha, Belo Horizonte, MG, Brazil

## Introduction

Brain death induces a massive inflammatory response. The majority of transplants are derived from donors who suffered from brain injury. The possible relation of clinical profile and cytokines in donors has been poorly explored. The objectives of this study were to analyze clinical characteristics of brain-dead donors and the correlation with cytokine profile in the ICU of a unique tertiary-care hospital.

## Methods

We evaluated 120 consecutive potential brain-dead organ donors (mean age 34.9 years, 74.2% males) between July 2007 and June 2008. Plasma cytokines (TNF, IL-2, IL-4, IL-5, IL-6, IL-8, IL-10, IFNγ) were measured in 40 donors immediately after criteria for brain death (or confirmatory tests) and after obtaining consent from families. Cytokines were assessed by cytometric bead array in the plasma and all laboratory personnel were blinded to clinical information.

## Results

The main cause of brain death was cerebral trauma (80%) and cerebral vascular accidents. The use of vasoactive agents was 90.6%. The median time of stay in the ICU was 2 days and the mean of organs transplanted was 2.2. Data (mean pg/ml) of cytokines were: IL-2, 3.32; IL-4, 2.63; IL-5, 11.4; IL-10, 25.99; IFN, 9.72; and TNF, 2.32. In 35% of donors IL-6 was above 5,000 pg/ml and in 15% IL-8 was below the detection limit of analysis. We did not find correlation (nonparametric statistical tests) between cytokines and gender, age, and laboratory tests of our organ donors. Pearson correlation between IL-6 and TNF was 0.001. IL-2 and IL-4, IL-5, IL-10 and IFN presented Pearson correlation ≤0.00. See Table 1.

**Table 1:** Cytokine levels

Cytokine	*P *value
IL-2	0.147
IL-4	0.044
IL-5	0.252
IL-8	0.764
IL-10 Th2	0.214
IFNγ	0.001

## Conclusion

Levels of proinflammatory and anti-inflammatory cytokines were increased in brain-dead donors and were correlated. There was no difference between cytokines and clinical and laboratory profiles.
